# Sorafenib inhibits macrophage-mediated epithelial-mesenchymal transition in hepatocellular carcinoma

**DOI:** 10.18632/oncotarget.9438

**Published:** 2016-05-18

**Authors:** Yan-Ru Deng, Wen-Bin Liu, Zhe-Xiong Lian, Xingsheng Li, Xin Hou

**Affiliations:** ^1^ Intensive Care Unit, Affiliated Provincial Hospital of Anhui Medical University, Hefei, China; ^2^ Department of Hepatic Surgery and Anhui Province Key Laboratory of Hepatopancreatobiliary Surgery, Affiliated Provincial Hospital of Anhui Medical University, Hefei, China; ^3^ Liver Immunology Laboratory, Institute of Immunology and School of Life Sciences, University of Science and Technology of China, Hefei, China; ^4^ Department of Gerontology, The Second Affiliated Hospital of Chongqing Medical University, Chongqing, China; ^5^ Anhui Provincial Laboratory of Microbiology and Parasitology, Department of Microbiology and Parasitology, Anhui Medical University, Hefei, Anhui, China

**Keywords:** hepatocellular carcinoma, epithelial-mesenchymal transition, sorafenib, HGF, Met

## Abstract

Tumor-associated macrophages, crucial components of the microenvironment in hepatocellular carcinoma, hamper anti-cancer immune responses. The aim of the present study was to investigate the effect of sorafenib on the formation of the tumor microenvironment, especially the relationship between polarized macrophages and hepatocytes. Macrophage infiltration was reduced in patients with hepatocellular carcinoma who were treated with sorafenib. *In vitro*, sorafenib abolished polarized macrophage-induced epithelial mesenchymal transition (EMT) and migration of hepatocellular carcinoma cells but not normal hepatocytes. Moreover, sorafenib attenuated HGF secretion in polarized macrophages, and decreased plasma HGF in patients with hepatocellular carcinoma. Additionally, sorafenib abolished the polarized macrophage-induced activation of the HGF receptor Met in hepatocellular carcinoma cells. Our findings suggest that sorafenib inhibits polarized macrophage-induced EMT in hepatocellular carcinoma cells via the HGF-Met signaling pathway. These results contribute to our understanding of the immunological mechanisms that underlie the protective effects of sorafenib in hepatocellular carcinoma therapy.

## INTRODUCTION

Hepatocellular carcinoma is the fifth most commonly occurring solid tumor worldwide, with relatively high and increasing incidence as well as frequent relapse and dismal prognosis [1]. Tumor-associated macrophages located in hepatocellular carcinomas increase tumor recurrence after liver resection and reduce patient survival [2, 3]. Tumor-associated macrophages represent polarized macrophages that are opposed to the actions of the pro-inflammatory macrophages [4]. Hence, interference with macrophage polarization may shift macrophage function from cancer facilitating to cancer suppressing, suggesting a potential approach for clinical therapy.

Sorafenib (Nexavar) is the first oral multikinase inhibitor well-known for its influence on tumor signaling and vasculature. This compound was recently approved for use in hepatocellular carcinoma [5]. Sorafenib blocks receptor tyrosine kinase signaling and inhibits downstream Raf serine/threonine kinase activity, thereby inhibiting the proliferation and survival of tumor cells [6]. However, the effect of sorafenib on the interaction between macrophages and hepatocytes remains elusive.

Epithelial-mesenchymal transition (EMT) is a developmental cellular program in which polarized epithelial cells lose epithelial properties, reduce intercellular adhesions, and acquire mesenchymal characteristics [7]. This phenotypic change is important in the development of the invasive and metastatic potentials of cancer progression [8–10]. Generally, a wide variety of factors can stimulate EMT progression, including transforming growth factor-β (TGF-β), hepatocyte growth factor (HGF), and interleukin-6 (IL-6). Among these cytokines, TGF-β is considered to be the most important activator of EMT, and IL-6 can interact with TGF-β to facilitate EMT [11, 12]. Through activating the Met signaling pathway, HGF (also known as a scattering factor) modifies the tumor microenvironment to facilitate cancer progression [13].

In this study, we found that macrophage infiltration was reduced in patients with hepatocellular carcinoma who were administered sorafenib. Sorafenib suppressed polarized macrophage-induced EMT and cellular migration of hepatocellular carcinoma cells. After treatment with sorafenib, HGF secretion was decreased in macrophages, which reduced the polarized macrophage-conditioned medium-induced activation of HGF-Met signaling in hepatocellular carcinoma cells but not in normal liver cells. In addition, patients with hepatocellular carcinoma also showed decreased HGF concentrations after sorafenib treatment. The discrepant reaction of hepatocellular carcinoma cells and normal liver cells to polarized macrophage-conditioned medium might be due to different expression levels of the HGF receptor Met. These results contribute to elucidating the immunological mechanisms that lead to the protective effects of sorafenib in hepatocellular carcinoma therapy.

## RESULTS

### Effects of sorafenib therapy in a representative patient with hepatocellular carcinoma

**Figure 1:**
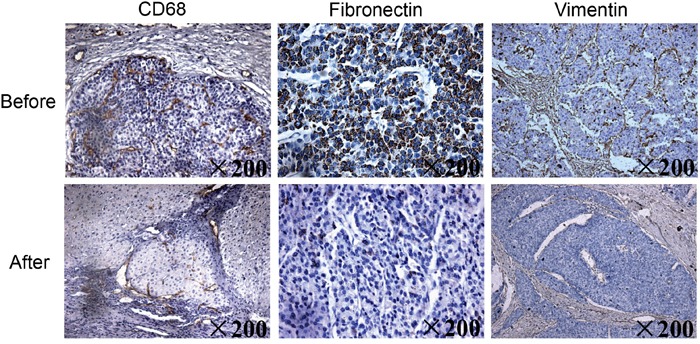
Hepatic biopsy samples obtained from a patient with hepatocellular carcinoma before and after 30 weeks of sorafenib therapy Immunohistochemistry stained liver samples were obtained before and after sorafenib therapy to determine the expression of CD68^+^ infiltrating macrophages and the EMT-related proteins fibronectin and vimentin (×200).

A 65-year-old man with hepatocellular carcinoma received sorafenib therapy. After thirty weeks of treatment, a computed tomography scan showed that the size of his tumor had shrunk. We performed paired liver biopsies (i.e., before and after sorafenib treatment) in this patient to evaluate changes in the infiltration of macrophages. A histological examination showed that the number of tumor-infiltrating CD68^+^ macrophages was reduced after sorafenib therapy. In addition, the expression levels of the EMT-related proteins fibronectin and vimentin were also decreased after sorafenib therapy (Figure 1).

### Sorafenib suppresses polarized macrophage-induced EMT in hepatocellular carcinoma cells

To elucidate the influence of sorafenib on macrophages, we stimulated the human monocyte cell line THP1 with PMA, and then seeded the cells on 12-well plates for 24 h to generate polarized macrophage. Real-time PCR was performed to analyze pro-apoptotic genes in polarized macrophages treated with or without sorafenib. The results showed that sorafenib treatment did not increase pro-apoptotic gene expression in polarized macrophages (Supplementary Figure 1). These data demonstrated that 3 h of sorafenib treatment would not suppress the survival of polarized macrophages.

**Figure 2:**
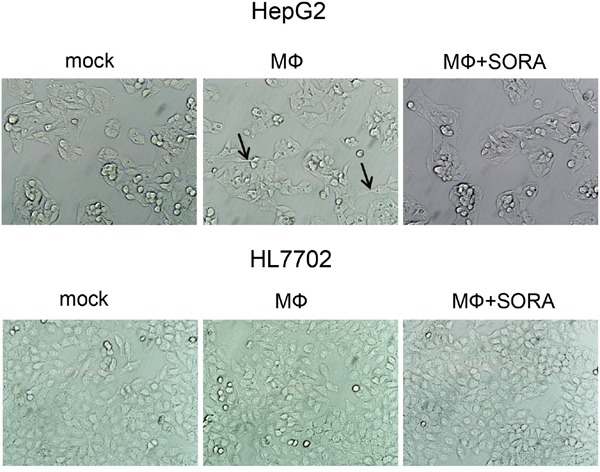
Sorafenib treatment counteracts polarized macrophage-induced EMT in HepG2 cells THP1 cells were stimulated with PMA for 3 h, washed twice to remove PMA, and seeded on 12-well plates (1 × 10^6^ cells/well) for 24 h to generate polarized macrophages. Sorafenib or DMSO (mock) was added to cells for 3 h. This treatment was followed by a medium exchange and stimulation with LPS (1 ng/mL) for 24 h. HepG2 and HL7702 cells were photographed and cell morphology was evaluated 48 h after culturing with the supernatant of polarized macrophages. Polarized macrophage (MФ) culture conditions: mock, DMSO; MФ, DMSO + LPS; MФ + SORA, sorafenib (1.2 μg/mL) + LPS. Arrows indicate elongated cells.

We then cultured the human liver carcinoma cell line HepG2 and the normal human liver cell line HL7702 with the supernatant from polarized macrophages. After 48 h, HepG2 cells exhibited morphological changes characteristic of EMT in which the scattering of the cells increased and their shape elongated. By contrast, the morphological changes in HL7702 cells were insignificant. More importantly, after treatment with sorafenib, the supernatant from polarized macrophages did not alter the morphology of HepG2 cells (Figure 2).

**Figure 3:**
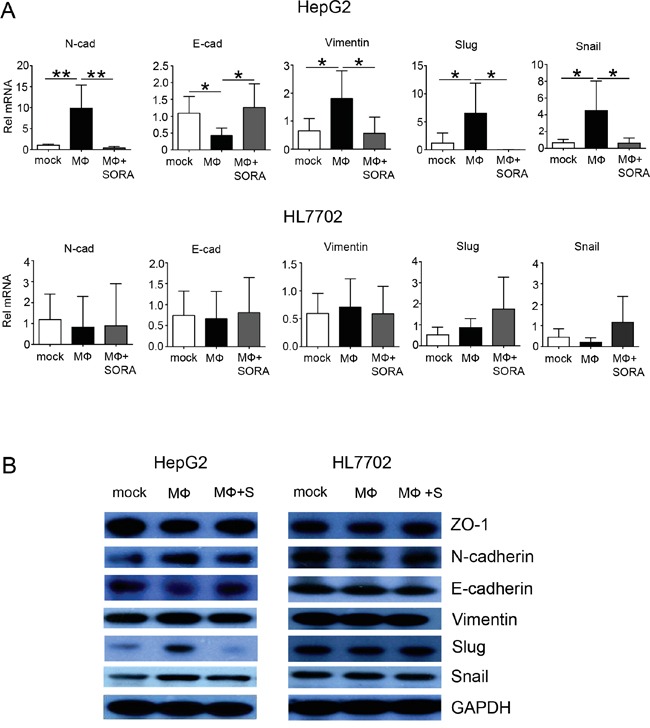
Sorafenib inhibits polarized macrophage-induced EMT-related gene and protein expression in HepG2 cells HepG2 and HL7702 cells (2 × 10^5^ cells/well) were seeded in 6-well tissue culture dishes. After the cells incubated for 24 h, the medium was exchanged with the supernatant of polarized macrophages (MФ) that had been stimulated under the following conditions: mock, DMSO; MФ, DMSO + LPS; MФ + SORA, sorafenib (1.2 μg/mL) + LPS. HepG2 and HL7702 cells were collected 48 h after the medium exchange. **A.** Quantitative PCR analysis of relative mRNA levels (Rel mRNA) of EMT-related genes *N-cad, E-cad, Vimentin, Snail*, and *Slug* in HepG2 and HL7702 cells. Values are normalized to the housekeeping gene *Gapdh* in the same sample and expressed as fold change in comparison with mock group. Data are expressed as mean ± SD, *P < 0.05; **P < 0.01. **B.** Western blot analysis of EMT-related proteins vimentin (D21H3), N-cadherin, ZO-1 (D1D12), Snail (C15D3), Slug (C19G7), and E-cadherin. GAPDH was used as a loading control.

Along with these morphological alterations, the mRNA expression of the EMT-related genes *Vimentin, Snail*, and *Slug* decreased in HepG2 cells when polarized macrophages were pretreated with sorafenib (Figure 3A) [14]. Furthermore, the supernatant from polarized macrophages pretreated with sorafenib did not increase the mRNA expression of EMT-related genes in HL7702 cells (Figure 3A). Cadherin switching, which is characterized by the downregulation of E-cadherin and upregulation of N-cadherin, is one of the most important features of EMT [15]. Sorafenib treatment inhibited mRNA expression of N-cadherin, and the mRNA level of E-cadherin was upregulated. These data suggest that sorafenib treatment suppresses the cadherin switching that was induced by polarized macrophages.

Consistent with the mRNA changes, the supernatant from polarized macrophages decreased protein expression levels of two epithelial markers (the adherens junction protein E-cadherin and the tight junction protein ZO-1) in HepG2 cells, whereas the expression levels of the intermediate filament proteins vimentin, E-cadherin regulation proteins Snail and Slug, and N-cadherin were upregulated. These effects were reversed when polarized macrophages were pretreated with sorafenib (Figure 3B and statistical analysis in Supplementary Figure 2). Additionally, EMT-related mRNA and protein expression were not notably changed in HL7702 cells cultured with the supernatant from polarized macrophages treated or untreated with sorafenib. These data indicate that polarized macrophage-induced EMT is suppressed by sorafenib only in hepatocellular carcinoma cells.

### Sorafenib inhibits polarized macrophage-induced cellular migration of hepatocellular carcinoma cells

**Figure 4:**
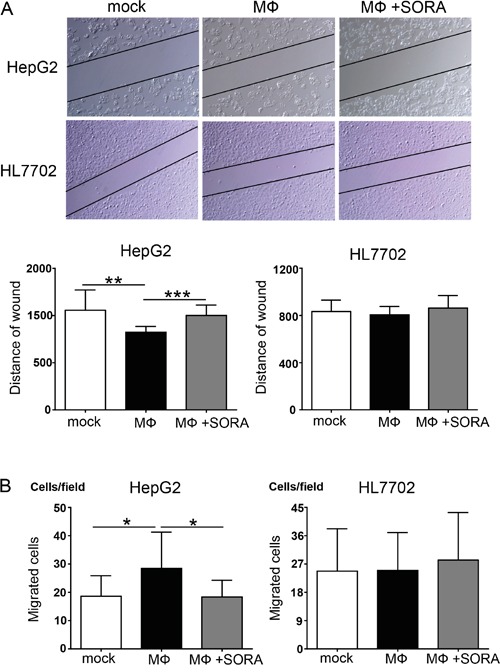
Polarized macrophages pretreated with sorafenib inhibit cellular migration of HepG2 cells **A.** SubconfluentHepG2 and HL7702 cells were scratched with a plastic pipette tip and incubatedwith the supernatant of polarized macrophages (MФ) that were stimulated under the following conditions: mock, DMSO; MФ, DMSO + LPS; MФ + SORA, sorafenib (1.2 μg/mL) + LPS. The results of this wound healing assay were photographed and measured. **B.** HepG2 and HL7702 cells transmigrate toward sorafenib-treated polarized macrophage cultures. *P < 0.05, **P < 0.01, and ***P < 0.001 for the indicated comparisons.

The data above demonstrated that sorafenib inhibited polarized macrophage-induced EMT in hepatocellular carcinoma cells. We next investigated whether the influence of sorafenib on polarized macrophages leads to an inhibition of the cellular migration of hepatocellular carcinoma cells. As shown in Figure 4A, the results of the wound healing assay revealed that stimulation of polarized macrophages increased the cellular migration of HepG2 cells but not of HL7702 cells. However, the cellular migration of HepG2 cells was significantly decreased when macrophages were pretreated with sorafenib, and this effect was not observed in HL7702 cells (Figure 4A). Furthermore, transwell experiments revealed that polarized macrophages stimulation increased the number of migrated HepG2 cells, and this effect could be blocked by pretreating macrophages with sorafenib (Figure 4B). As before, the same effects were not observed in HL7702 cells. These results suggest that sorafenib inhibits the macrophage-induced cellular migration of hepatocellular carcinoma cells.

### Sorafenib changes cytokine production in polarized macrophages

**Figure 5:**
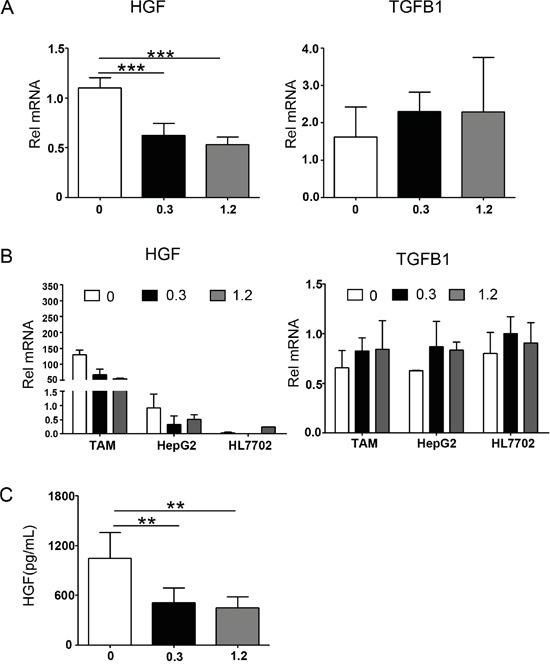
Cytokine profiles in a transwell system containing polarized macrophages, HepG2, and HL7702 cells Polarized macrophages (12-well plate, 1 × 10^6^ cells/well) were treated with sorafenib or DMSO (mock) for 3 h. The medium was then changed and stimulated with LPS (1 ng/mL) for 24 h. Transwells (0.4 μm pores) containing 1 × 10^5^ HepG2 or HL7702 cells were subsequently placed on top of the cultured macrophages for 24 h. **A.** Quantitative PCR analysis of relative mRNA (Rel mRNA) levels of HGF and TGF-β1/TGFB1 in polarized macrophages. Results were expressed as fold amplification over 0 group following normalization with *Gapdh*. Data were expressed as mean ± SD, ***P < 0.001. **B.** Comparison of mRNA expression of HGF and TGF-β1/TGFB1 in polarized macrophages, HepG2, and HL7702 cells. Results were expressed as fold amplification over HepG2 0 group following normalization with *Gapdh*. Data were expressed as mean ± SD. **C.** Polarized macrophages were treated with sorafenib or DMSO (mock), and then stimulated with LPS (1 ng/mL) for 24 h. The secretion of cytokines into the culture supernatants was determined by ELISA. Polarized macrophage cultured conditions: 0, DMSO + LPS; 0.3, sorafenib (0.3 μg/mL) + LPS; 1.2, sorafenib (1.2 μg/mL) + LPS. **P < 0.01.

We also analyzed cytokine secretion of polarized macrophages, which could stimulate the EMT progression. Changes in the mRNA expression of EMT-related cytokines in macrophage treated with or without sorafenib were evaluated by real-time PCR. Compared with untreated controls, sorafenib markedly inhibited mRNA expression of HGF without significantly decreasing the mRNA expression of TGF-β1 (Figure 5A). However, changes in other EMT-related cytokines, EGF, IL-10, and IL-6, were not consistent with the morphologic changes occurring during EMT (data not shown).

Because HGF and TGF-β1 can be secreted not only by macrophages but also by hepatocytes, the mRNA expression levels of HGF and TGF-β1 in HepG2 and HL7702 cells were also evaluated. As shown in Figure 5B, the mRNA expression of HGF in macrophages was 143-fold higher than that in HepG2 cells and 3,232-fold higher than that in HL7702 cells. However, the differences in TGF-β1 mRNA expression between macrophages and hepatocytes (HepG2 and HL7702 cells) were not remarkable (Figure 5B). We also used an ELISA to analyze the HGF protein expression level in the macrophage-conditioned medium. These results were consistent with those for the HGF mRNA expression (Figure 5C). Based on these results, we concluded that sorafenib inhibits the HGF secretion of polarized macrophages.

### Sorafenib therapy effects in patients with hepatocellular carcinoma

**Figure 6:**
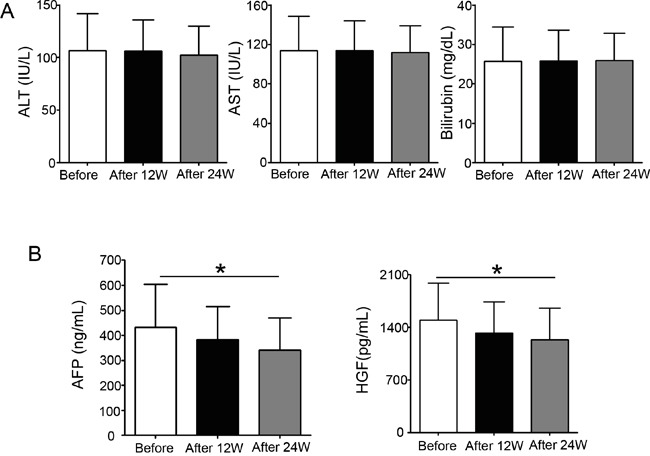
Sorafenib therapy reduces plasma HGF and alpha-fetoprotein concentrations in patients with hepatocellular carcinoma The plasma from patients with hepatocellular carcinoma was collected before and after sorafenib therapy. **A.** Alanine transaminase, (ALT), aspartate aminotransferase (AST), and bilirubin levels were analyzed before and 12 or 24 weeks after sorafenib therapy. **B.** Alpha-fetoprotein and HGF concentrations were determined before and after 12 or 24 weeks of sorafenib treatment. *P < 0.05.

To verify the results we obtained from *in vitro* experiments, we collected plasma from patients before and after sorafenib therapy. Table 1 showed the clinical and laboratory findings of patients with hepatocellular carcinoma who received sorafenib therapy for 12 and 24 weeks. A statistical analysis revealed that 12 or 24 weeks of sorafenib therapy did not alter alanine transaminase, aspartate aminotransferase, and bilirubin levels (Figure 6A), and 12 weeks of sorafenib therapy had no significant inhibition on alpha-fetoprotein (AFP) and HGF. However, after 24 weeks of therapy, the concentration of HGF in patient plasma was markedly reduced, which is consistent with the decrease also observed in AFP (Figure 6B). These results demonstrate that sorafenib therapy inhibits HGF secretion in patients with hepatocellular carcinoma.

**Table 1:** Clinical and Laboratory Findings of the Patients with Hepatocellular Carcinoma Accepted Sorafenib Therapy

No.	Gender	Age	ALT (IU/L)			AST (IU/L)			Bilirubin (μmol/L)			AFP (ng/mL)			HGF (ng/mL)		
			Before	After 12W	After 24W	Before	After 12W	After 24W	Before	After 12W	After 24W	Before	After 12W	After 24W	Before	After 12W	After 24W
1	M	72	134	126	118	148	138	125	29.86	25.78	27.54	356	299	221	1060	760	690
2	M	73	112	117	119	124	131	137	19.65	21.52	20.43	278	324	289	1450	1030	890
3	M	61	89	82	87	96	90	99	16.80	17.31	18.43	321	456	378	1310	1290	1070
4	F	54	157	146	141	148	139	131	20.65	19.98	18.65	753	574	432	2980	2050	1780
5	M	63	107	115	111	102	121	116	31.89	29.57	27.68	401	332	278	1950	1670	1890
6	M	59	96	102	95	113	124	120	26.13	28.67	27.93	299	213	167	1780	2070	2210
7	M	65	171	158	147	169	152	157	35.72	39.45	37.81	534	327	287	1930	1670	1450
8	M	62	81	95	87	92	108	98	24.78	26.83	25.56	356	301	325	1210	970	1040
9	M	71	96	112	107	105	117	121	28.93	34.78	35.72	364	314	243	1080	1240	1320
10	F	48	128	135	132	139	143	152	24.97	28.59	26.93	447	385	356	2010	1650	1340
11	M	53	136	127	131	142	120	127	26.78	25.73	27.49	621	592	504	1720	1320	1280
12	F	68	103	99	95	114	107	100	19.73	18.94	21.67	406	428	367	1630	1580	1190
13	M	43	56	63	58	65	70	74	15.75	16.72	18.72	275	248	197	1470	1520	1640
14	M	45	81	92	86	94	99	91	21.94	19.64	24.75	307	265	186	1210	1320	1290
15	M	67	73	68	71	87	92	89	17.81	18.34	21.51	374	407	398	1690	1310	1060
16	F	61	132	127	125	146	152	141	32.76	29.67	27.43	584	498	356	1850	1560	1430
17	F	56	147	151	142	162	153	160	21.62	23.56	24.93	625	629	567	1320	1160	980
18	F	67	164	129	120	172	147	130	39.87	35.82	30.76	471	389	456	2050	1780	1530
19	M	49	178	158	153	181	169	157	45.35	40.71	38.59	871	643	521	1750	1690	1580
20	F	45	97	103	95	106	119	115	27.48	28.94	30.43	369	428	336	1530	1440	1320
21	M	69	62	59	60	69	72	76	16.95	17.69	14.83	175	131	125	670	750	720
22	M	54	128	116	103	135	118	109	39.67	37.81	33.57	561	535	595	1430	1520	1570
23	M	74	136	142	130	146	158	140	45.83	41.57	39.32	711	524	561	1640	1460	1520
24	M	81	56	61	67	63	59	68	15.96	18.01	17.43	342	456	489	2010	1740	1690
25	M	46	80	78	71	86	89	95	17.95	19.89	21.46	574	365	313	1320	1350	1240
26	F	62	82	87	93	75	91	93	21.83	20.02	23.74	329	287	241	1060	890	670
27	F	57	92	102	98	64	78	81	29.73	31.25	35.84	481	385	351	1530	1240	980
28	M	45	67	65	60	81	76	80	15.82	16.36	19.67	123	163	189	720	560	430
29	M	67	85	87	79	94	90	83	20.85	23.56	19.45	275	305	242	590	430	560
30	M	74	73	79	84	85	91	87	16.72	17.83	18.87	386	267	232	980	780	640

### Sorafenib attenuates HGF-Met signaling in hepatocellular carcinoma cells previously activated by polarized macrophage-conditioned medium

**Figure 7:**
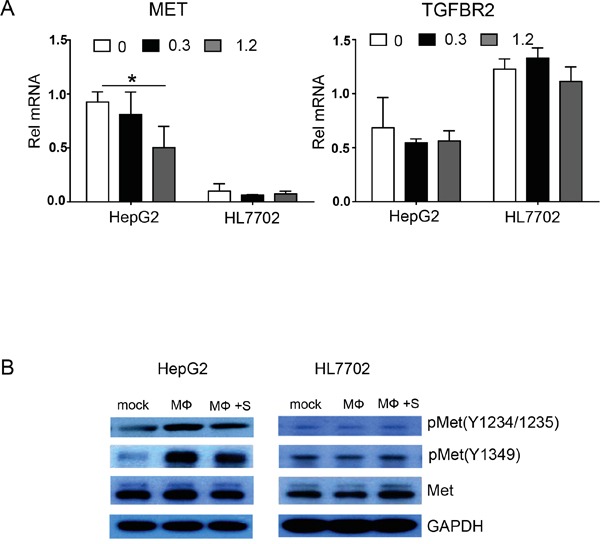
Polarized macrophages pretreated with sorafenib inhibit HGF-Met signaling in HepG2 cells **A.** HepG2 and HL7702 cells (2 × 10^5^ cells/well) were cultured with the supernatant of polarized macrophages that were stimulated under the following conditions: 0, DMSO + LPS; 0.3, sorafenib (0.3 μg/mL) + LPS; 1.2, sorafenib (1.2 μg/mL) + LPS. Quantitative PCR analysis of relative mRNA (Rel mRNA) expression levels for the HGF receptor MET and the TGF-β1 receptor TGFBR2 in HepG2 and HL7702 cells. Data were expressed as fold amplification over HepG2 0 group following normalization with *Gapdh*. Data were expressed as mean ± SD, *P < 0.05. **B.** HepG2 and HL7702 cells were cultured with the supernatant of polarized macrophages (MФ) that were stimulated under the following conditions: mock, DMSO; MФ, DMSO + LPS; MФ + SORA, sorafenib (1.2 μg/mL) + LPS. HGF-Met signaling-related proteins pMet (Tyr1234/1235; D26), pMet (Tyr1349; 130H2), and Met (D1C2) were analyzed by western blot. GAPDH was used as a loading control.

Aberrant HGF-Met activation reportedly promotes tumor cell proliferation and metastasis via growth factor receptors and other oncogenic receptor pathways [13], and we found that sorafenib markedly inhibited HGF expression in polarized macrophages. To further investigate whether sorafenib inhibits macrophage-induced EMT and migration of hepatocellular carcinoma cells via the HGF-Met signaling pathway, we used real-time PCR to examine mRNA expression of HGF receptor Met and the TGF-β receptor TGFBR2 in HepG2 and HL7702 cells cultured with the supernatant from polarized macrophages. The results indicated that after being cultured with the supernatant of polarized macrophages, Met expression in HepG2 cells was 9.28-fold higher than that in HL7702 cells (Figure 7A). When polarized macrophages were pretreated with sorafenib, the expression of Met in HepG2 cells was significantly decreased. By contrast, this phenomenon was not observed in HL7702 cells (Figure 7A). We also examined TGFBR2 expression in HepG2 and HL7702 cells. The data showed that the expression of TGFBR2 in HL7702 cells was 1.79-fold higher than that in HepG2 cells. However, sorafenib treatment did not inhibit TGFBR2 expression in either HepG2 or HL7702 cells (Figure 7A).

We also used western blot analysis to examine the activation of HGF-Met signaling in HepG2 and HL7702 cells (Figure 7B and statistical analysis in Supplementary Figure 3). Met protein expression was not significantly altered in HepG2 cells regardless of whether the polarized macrophages were treated with sorafenib. Because the Met signaling pathway depends mainly on Met phosphorylation, we examined the expression of phosphorylated Met. The expression levels of phosphorylated Met at two sites, pMet Y1234/1235 and pMet Y1349, in HepG2 cells were increased after stimulation with the supernatant of polarized macrophages, and sorafenib pretreatment decreased the protein expression levels of pMet Y1234/1235 and pMet Y1349. By contrast, Met, pMet Y1234/1235, and pMet Y1349 protein expression levels were not significantly changed in HL7702 cells whether or not the polarized macrophages were treated with sorafenib. Taken together, these results indicate that sorafenib blocks only polarized macrophage-activated HGF-Met signaling in hepatocellular carcinoma cells.

## DISCUSSION

In this study, we investigated whether sorafenib could affect hepatocellular carcinoma microenvironment and alter the interaction between macrophages and hepatocytes. We found that sorafenib suppressed macrophage infiltration in a patient with hepatocellular carcinoma (Figure 1). We used *in vitro* experiments to demonstrate that polarized macrophage couldinduce EMT (Figure 2 and 3) and cellular migration (Figure 4) in HepG2 cells, which is a type of human hepatocellular carcinoma cell. This effect was not observed in HL7702 cells, a normal human liver cell, suggesting that sorafenib decreased the tumor microenvironment formation and inhibited the promoting effect of macrophages on EMT and migration of hepatocellular carcinoma cells. Moreover, the effect of sorafenib on the relationship between macrophages and normal hepatocytes was much smaller.

Previous studies have reported that macrophage-derived cytokines stimulate the progression of EMT. Thus, we examined the cytokines activating EMT in polarized macrophages. Among these cytokines, TGF-β1 is considered to be the most important activator of EMT, inducing EMT through various pathways [28, 37–40]. In addition, TGF-β1 can interact with other cytokines to regulate the progression of EMT, such as IL-6, IL-10, and EGF [12, 52–54]. Thus, we investigated the expression of these cytokines in polarized macrophages. However, the expression levels of IL-6, IL-10, and EGF were not consistent with the morphological changes in EMT (data not shown), and the expression of TGF-β1 was not significantly altered (Figure 5A). Only the secretion of HGF was consistent with EMT (Figure 5A and 5C). In addition, the mRNA expression level for HGF in macrophages was 100-fold higher than that in HepG2 or HL7702 cells (Figure 5B). These data revealed that HGF stimulated EMT through paracrine modes of action.

HGF is a pleiotropic growth factor originally isolated from rat platelets [55]. It is the most potent growth factor for hepatocytes, and its receptor Met is also expressed on normal hepatocytes [56]. Aberrant HGF-Met activation has been observed in many tumors, promoting cellular proliferation and metastasis via growth factor receptors and other oncogenic receptor pathways to facilitate cancer progression [13, 57]. The results of the present study demonstrated that sorafenib attenuated HGF secretion in polarized macrophages (Figure 5). Sorafenib also inhibited polarized macrophage-induced Met activation in hepatocellular carcinoma cells (Figure 7), suggesting that sorafenib inhibited macrophage-induced EMT and migration of hepatocellular carcinoma cells via the HGF-Met signaling pathway. The expression of Met was much lower in HL7702 cells than that in HepG2 cells (Figure 7B), which was one reason that polarized macrophages could not induce EMT and cellular migration in HL7702 cells.

The mRNA levels of HGF and Met in hepatocellular carcinoma are markedly increased compared with those in common liver cells [58]. A high serum HGF concentration is associated with poor prognosis after hepatic resection, and the serum HGF concentration represents the degree of the carcinogenic state of the liver in patients with chronic hepatitis C virus infection and cirrhosis [59–61]. Our findings showed plasma HGF and AFP concentrations were also reduced in patients receiving sorafenib therapy (Figure 6B), consistent with the results from our *in vitro* experiments. This effect of sorafenib, together with its anti-angiogenic activity, can contribute to additional clinical benefits against metastatic and aggressive phenotypes in patients with hepatocellular carcinoma.

## MATERIALS AND METHODS

### Patients and follow-up

The liver biopsy specimens were obtained from the Second Affiliated Hospital of Chongqing Medical University. The patients' plasma was from Affiliated Provincial Hospital of Anhui Medical University. The HCC diagnosis was according to the standard of American Association for the Study of Liver Diseases. The patient inclusion followed the following criteria: (a) diagnosed as advanced hepatocellular carcinoma; (b) computed tomography detection could define tumor morphology; (c) no other primary tumor; (d) no combination with other radiotherapy or chemotherapy. The initial dose of sorafenib was 800 mg daily, and the dosage was adjusted according to the patient's condition.

Informed consent was obtained from all subjects. The study protocol conformed to the ethical guidelines of the 1975 Declaration of Helsinki. We also obtained approval for this study from our institutional ethics committees.

### Cell lines

THP1, HepG2 cells were obtained from American Type Culture Collection (ATCC, Manassas, VA). HL7702 cells were obtained from Cell bank of Chinese Academy of Science (Shanghai, China). Cells were cultured according to the manufacturer's instructions.

### Cell culture and treatment

Human liver cell line HL7702 and human liver carcinoma cell line HepG2 were cultured in RPMI 1640 medium (Hyclone, Logan, UT, USA) with 10% fetal bovine serum (FBS), 100 units/ml penicillin, 100 mg/ml streptomycin medium (Hyclone), and incubated in a humidified incubator containing 5% CO2 at 37°C. Human monocyte cell line THP1 was stimulate with phorbol myristate acetate (PMA) (0.1μM, Invitrogen, Carlsbad, CA) for 3 hours, and then washed twice to remove PMA and seeded on 12-well plates (1×10^6^ cells/well) for 24 hours to generate polarized macrophages (Supplementary Figure 1). Sorafenib (Nexavar, BAY 43-9006, Germany) or DMSO (Sigma-Aldrich, St. Louis, MO, USA) carrier (mock) was added to cell culture for 3 hours. Treatment was followed by a medium exchange and stimulate with LPS (1ng/mlSigma-Aldrich, St. Louis, MO) for 24 hours. The supernatant of polarized macrophage was collected and centrifugated at 15,294×g for 15 min to remove cell debris.

### Wound healing assay

The method used for the wound healing assay has been previously described [62]. Briefly, the cells were plated onto 24-well plates and incubated in RPMI 1640 medium containing 10% FBS until they reached subconfluence. Wound healings were introduced to the subconfluent cell monolayer, using a plastic pipette tip. The cells were then cultured with the supernatant of polarized macrophage. After 24 hours, the wound healing area was photographed using a light microscope (IX71; Olympus). The wound distance from edge to edge was measured and averaged from 5 points per 1 wound area, using Imagine Pro Plus software. The 2 wound areas were evaluated in an experiment and the experiment was done in triplicate.

### Transwell and migration assay

Polarized macrophage (12-well plate, 1×10^6^ cells/well) treated with sorafenib or mock for 3 hours, and then the medium was exchanged. Transwells (0.4μm pores; Corning Inc., Corning, NY, USA) carrying 1×10^5^ HepG2 or HL7702 cells were subsequently placed on top of the cultured macrophage for 24 hours. Migration assays were modified by 8 μm pore transwells (Corning) carrying 2×10^4^ HepG2 or HL7702 cells. The cells to be analyzed (2×10^4^ cells/well) were seeded onto the upper chambers, and the upper chambers were placed into the lower chambers of 24-well culture dishes containing macrophage treated or untreated with sorefenib. After incubation for 36 hours, the media in the upper chambers were aspirated and the nonmigrated cells on the inner sides of the membranes were removed using a cotton swab. The cells that had migrated to the outer side of the membranes were stained with 0.1% crystal violet stain solution, and then counted using a light microscope. Migrated cells were averaged from 5 fields per 1 chamber and 3 chambers were used on 1 experiment.

### Quantitative real-time polymerase chain reaction (real-time qPCR)

Total RNA was extracted from cells using Trizol (Invitrogen, Carlsbad, CA). M-MLV Transcriptase (Invitrogen) was used for reverse transcription. Quantitative real-time PCR was performed using Premix Ex Taq (Takara, Japan). The expression levels of target genes were normalized to the housekeeping gene *Gapdh* and the results were calculated by ΔΔCt [63]. The primers used for real-time PCR are listed in Supplementary Table 1.

### Enzyme linked immunosorbent assay (ELISA)

The supernatant cytokines were quantified by ELISA for HGF (R&D Systems) according to the manufacturer's instructions.

### Morphologic analysis

HepG2 and HL7702 cells (2×10^5^ cells/well) were seeded in 6-well tissue culture dishes. Twenty-four hours after incubation, the medium was exchanged with the supernatant of polarized macrophage. After 48 hours, the cells were analyzed using a light microscope (IX71; Olympus).

### Western blot

Western blot analysis was performed as previously described [64]. Briefly, total cell lysates were prepared, and proteins were separated by SDS-PAGE and then transferred to the Immunobilon-P transfer membranes (Millipore, Billerica, Massachusetts). The membranes were washed, blocked, and incubated with the specific primary anti-human antibodies against Vimentin (D21H3), N-Cadherin, ZO-1(D1D12), Snail(C15D3), Slug(C19G7), E-Cadherin, pMet(Tyr1234/1235)(D26), pMet(Tyr1349)(130H2), Met(D1C2) and GAPDH, followed by incubation with HRP-labeled anti-rabbit secondary antibody (all from Cell Signaling Technology, Beverly, MA).

### Immunohistochemistry

Immunohistochemistry analysis was performed as described previously [64]. Primary anti-human antibodies against CD68 (PG-M1), Fibronectin and HRP labeled secondary antibodies were from Dako (Glostrup, Denmark); Primary antibody Vimentin (D21H3) was from Cell Signaling Technology (Beverly, MA).

### Statistical analysis

All data are presented as mean ± standard deviation (SD). Comparisons were made with Student's t test. All experiments were replicated two or three times, with similar results. *P* < 0.05 was considered statistically significant.

## SUPPLEMENTARY FIGURES AND TABLES


