# Supplementary material for: Sorafenib inhibits macrophage-mediated epithelial-mesenchymal transition in hepatocellular carcinoma
Source: Oncotarget. 2016 May 18;7(25):38292–305. doi: 10.18632/oncotarget.9438 (PMC5122390; doi:10.18632/oncotarget.9438)
Supplement: Supplementary file 1 [file oncotarget-07-38292-s001.pdf]

Sorafenib inhibits macrophage-mediated epithelial-mesenchymal transition in hepatocellular carcinoma

SUPPLEMENTARY FIGURES AND TABLE

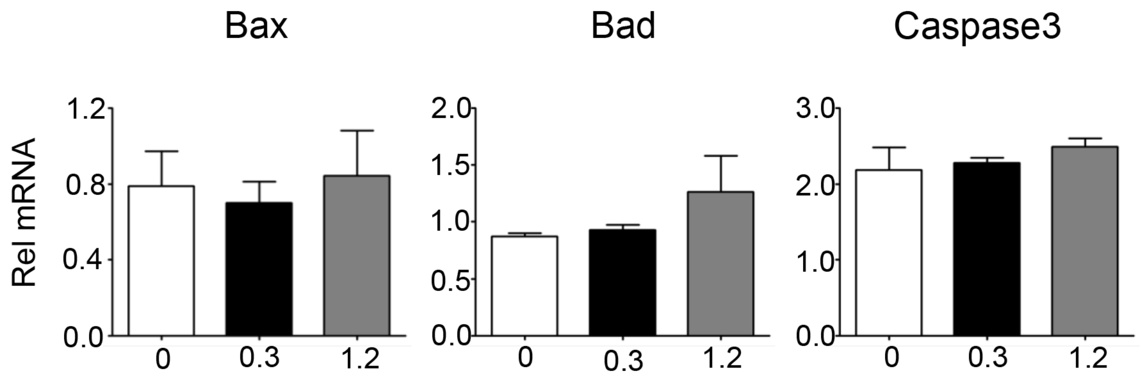

Supplementary Figure S1: The mRNA expression of pro-apoptotic genes in polarized THP1 macrophages treated or untreated with sorafenib. Sorafenib or DMSO vehicle (mock) was added to cultured cells for 3 h. This treatment was followed by a medium change and stimulation with LPS (1 ng/mL) for 24 h. Real-time PCR was performed to analyze the pro-apoptotic genes *Bax*, *Bad*, and *caspase3* in polarized THP1 macrophages treated or untreated with sorafenib. Polarized macrophage culture conditions: 0, DMSO + LPS; 0.3, sorafenib (0.3 µg/mL) + LPS; 1.2, sorafenib (1.2 µg/mL) + LPS.

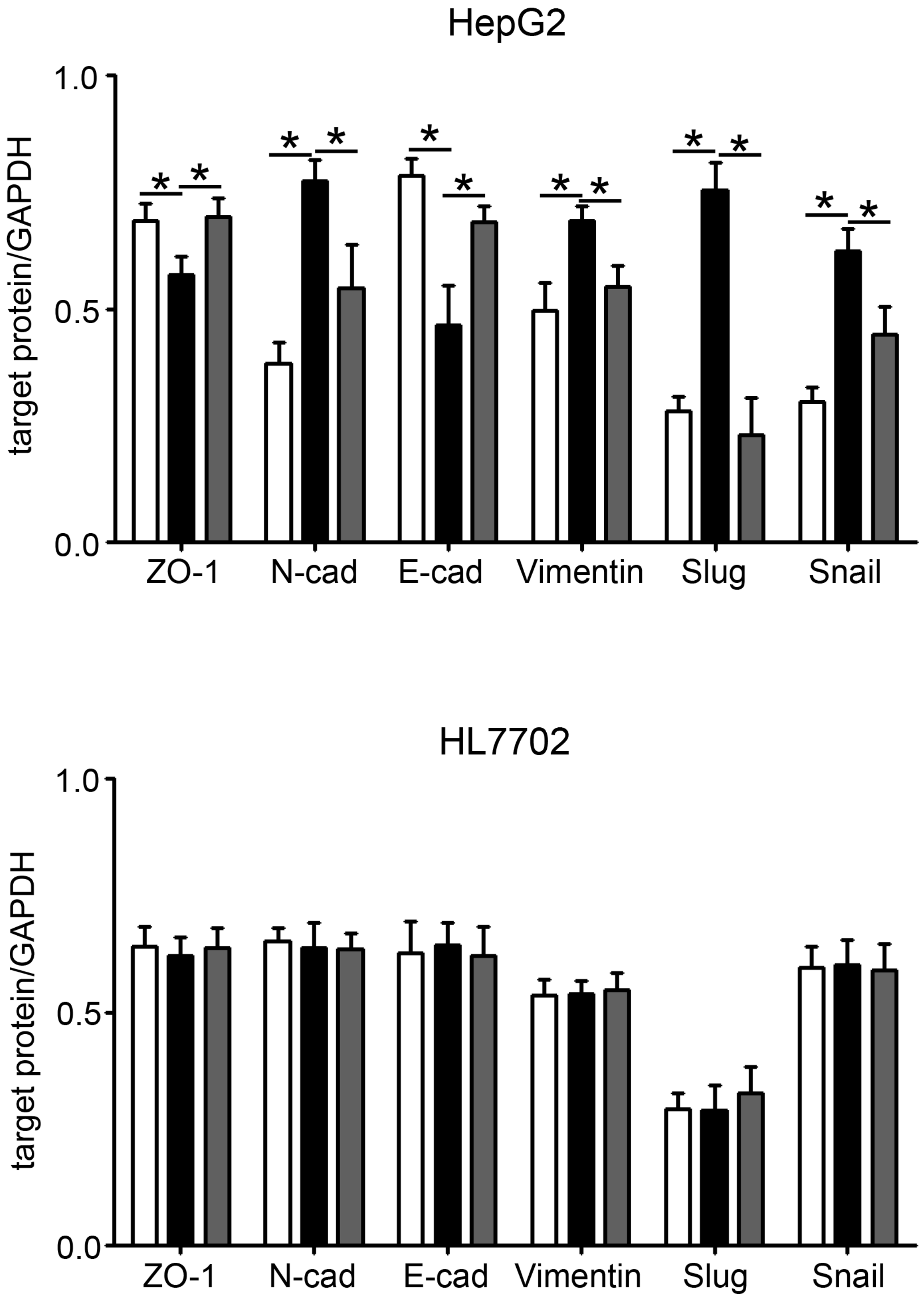

Supplementary Figure S2: Relative expression levels of ZO-1 (D1D12), N-cadherin, E-cadherin, vimentin (D21H3), Snail (C15D3) and Slug (C19G7). HepG2 and HL7702 cells were incubated with the supernatant of polarized macrophages (MΦ) that had been stimulated under the following conditions: mock, DMSO; MΦ, DMSO + LPS; MΦ + SORA, sorafenib (1.2 μg/mL) + LPS. HepG2 and HL7702 cells were collected 48 h later, and subjected to Western blot analyses (Figure 3B). Densitometry of the intensities of the protein band on a series of Western blots was used to quantify the levels of each protein expression. Data were presented as the expression levels relative to the GAPDH levels in each group. Data were from 3 to 4 separate experiments and presented as mean ± SD. $^*P < 0.05$.

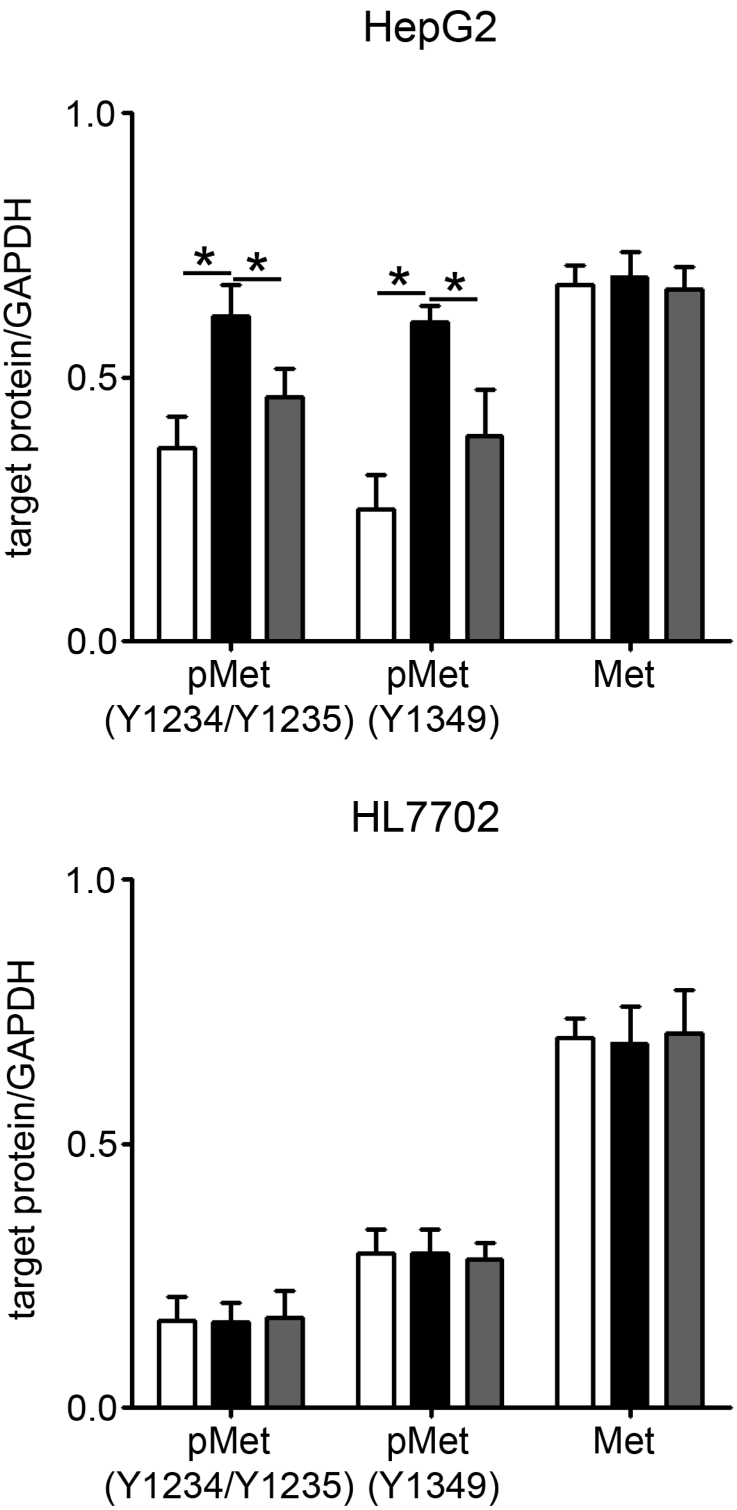

Supplementary Figure S3: Relative expression levels of pMet (Tyr1234/1235; D26), pMet (Tyr1349; 130H2), and Met (D1C2). HepG2 and HL7702 cells were cultured with the supernatant of polarized macrophages (MΦ) that were stimulated under the following conditions: mock, DMSO; MΦ, DMSO + LPS; MΦ + SORA, sorafenib (1.2 μg/mL) + LPS. pMet (Tyr1234/1235; D26), pMet (Tyr1349; 130H2), and Met (D1C2) were detected by western blot (Figure 7B). Data were presented as the expression levels relative to the GAPDH levels in each group based on densitometry of the intensities of protein bands. Data were from 3 to 4 separate experiments and presented as mean ± SD. $*P < 0.05$.

Supplementary Table S1: Primers used for quantitative PCR

Genes	*Forward (5′-3′)*	*Reverse (5′-3′)*
HGF	GTTCAATGTGGGACAAGAACATGG	GGATTTCGGCAGTAATTCTCATTCA
MET	CCTCACCATAGCTAATCTTGGGACA	CACAATCACTTCTGGAGACACTGGA
TGFB1	TCGACATGGAGCTGGTGAAA	GGGACTGGCGAGCCTTAGTT
TGFBR2	GCTTCACTCTGGAAGATGCC	AAGGAGTGTGGTCACTGTGC
Vimentin	TGAGTACCGGAGACAGGTGCAG	TAGCAGCTTCAACGGCAAAGTTC
E-cad	TTAAACTCCTGGCCTCAAGCAATC	TATCTTGGGCAAAGCAACTG
Snail	CTTGTGTCTGCACGACCTGT	CTTCACATCCGAGTGGGTTT
Slug	GCACTGTGATGCCCAGTCTA	CAGTGAGGGCAAGAGAAAGG
N-cad	CGAATGGATGAA AGA CCCATCC	GGAGCCACTGCCTTCATA GTC
Bax	GCTCTGAACAGATCATGAAG	GATGGTCACTGTCTGCCATG
Bad	AGAGTATGTTCCAGATCCCAG	GTCCTCGAAAAGGGCTAAGC
Caspase3	AGTCAGTGGACTCTGGGATC	GTACAGTTCTTTCGTGAGCA
Gapdh	CCATGTTCGTCATGGGTGTGAACCA	GCCAGTAGAGGCAGGGATGATGTTC

All the sequences of target genes were got from NCBI GenBank. The primes were designed and synthesized by Takara Biotechnology (Dalian) Corporation.
